# Understanding the Remplissage: History, Biomechanics, Outcomes, and Current Indications

**DOI:** 10.1007/s12178-024-09900-3

**Published:** 2024-05-20

**Authors:** Ryan Freshman, Benjamin Lurie, Grant Garcia, Joseph Liu

**Affiliations:** 1 USC Epstein Family Center for Sports Medicine at Keck Medicine of USC, Los Angeles, CA 90033 USA; 2 Proliance Surgeons Orthopedic Specialists of Seattle, 2409 N. 45Th Street, Seattle, WA 98103 USA

**Keywords:** Remplissage, Shoulder instability, Bankart, Labral tear, Shoulder dislocation, Recurrent instability, Capsulorraphy

## Abstract

**Purpose of Review:**

Arthroscopic remplissage has continued to gain popularity as an adjunct to Bankart repair for patients with anterior shoulder instability. Although the original remplissage technique was described over 15 years ago, our understanding of when and how to use this procedure continues to evolve. This article provides a review of how remplissage affects shoulder biomechanics, compares clinical outcomes between remplissage and other procedures for shoulder instability, and discusses current indications for remplissage.

**Recent Findings:**

Current research focuses on the use of remplissage across a wide range of glenoid bone loss. Remplissage appears effective at preventing recurrent instability in patients with glenoid bone loss up to 15% of the glenoid width. However, once glenoid bone exceeds 15%, outcomes tend to favor bony reconstruction procedures such as Latarjet. Results of biomechanical studies examining shoulder range of motion (ROM) after remplissage are mixed, though clinical studies tend to report no significant limitations in ROM when remplissage is added to a Bankart repair.

**Summary:**

Adding a remplissage to conventional Bankart repair may improve clinical outcomes and lower rates of recurrent instability without significantly altering shoulder ROM. However, surgeons should recognize its limitations in treating patients with large amounts of glenoid bone loss and should be prepared to discuss alternative procedures on a case-by-case basis. Absolute indications and contraindications for remplissage are not well defined currently and require further scientific research.

## History and Background

The arthroscopic remplissage procedure (French for “filling”) for anterior shoulder instability involves filling the Hill-Sachs impaction fracture on the posterior humeral head with the overlying infraspinatus tissue and posterior capsule. Remplissage was first described as an adjunctive open procedure by Connolly [13] et. al. in 1972 to address large Hill-Sachs lesions (HSLs), which were identified as early as 1890 as a cause of shoulder instability and formally classified by Hill and Sachs in 1940 [29]. Multiple techniques had been previously utilized to address significant Hill-Sachs defects, including osteochondral allograft transplantation, open capsular shift, rotational proximal humeral osteotomy and humeral head disimpaction and grafting. With the advent of remplissage, these techniques have been largely abandoned and are typically utilized only for very large HSLs due to increased levels of technical difficulty, higher complication rates, and relative success in treating significant bipolar bone loss with isolated bony reconstruction of the glenoid. However, in situations where bipolar bone loss is present to a lesser degree, remplissage has quickly become the preferred method to manage relevant HSLs in an arthroscopic manner, and can be used conjunction with a Bankart repair (BR), Latarjet or other glenoid augmentation procedures [10].

The modern technique for arthroscopic remplissage was first described by Wolf, Pollack and Smalley [57] in 2007 and further detailed by Purchase et. al. [50] in 2008. Two suture anchors are placed in the freshened HSL and then tied independently over the posterior capsule and infraspinatus tendon. Wolf and Arianjam went on to publish their series of 45 patients with bipolar bone loss and an engaging HSL. [56] At 2–10 year follow-up, they found a found a recurrence rate of 4.4% despite all patients having at least some degree of glenoid bone loss, and reported no clinically significant loss of external rotation.

The initial indication for remplissage was for treatment of the engaging HSL with less than 25% glenoid bone loss [3, 4, 49, 56]. The concept of dynamic engagement was described by Burkhart and De Beer [5], and has remained a validated and widely utilized tool to identify HSLs that require treatment. Dynamic engagement between the HSL and the anterior rim of the glenoid can be visualized arthroscopically prior to BR. Burkhart and De Beer clarified that while there is always some position of the shoulder where the HSL will engage the glenoid, this engagement is only clinically significant if it occurs with the arm in a functional athletic position. This treatment paradigm was supported by Garcia et. al., who found that when large, engaging HSL were treated with BR alone, there was a 57.1% recurrence of instability compared to 20.0% when remplissage was added [23].

While arthroscopic assessment of engagement is an excellent indicator of the need for remplissage and has been shown to lower dislocation rates in this high risk group [30, 42••], it relies on surgeon interpretation and may be affected by joint laxity, muscle paralysis and capsular distension. The glenoid track concept developed by Yamamoto et. al. [58] provides a quantitative, preoperative formula to determine whether a HSL will engage the anterior glenoid and increase the likelihood of failure of isolated BR. This concept was further developed by Di Giacomo, Itoi and Burkhart [15], who proposed that ‘off-track’ HSLs require treatment either by addressing deficiency of the glenoid, humerus, or both. The glenoid track (GT) is defined by the equation GT = 0.83D-d, where D is the diameter of the glenoid and d is the amount of anterior bone loss present. The GT is compared to the Hill-Sachs interval (HSI), which is defined as the distance from the medial aspect of the HSL to the medial edge of the rotator cuff insertion. If the value of the GT is greater than the HSI, the lesion is considered to be “on-track” and at low risk of dislocation. Conversely, if the value of the HSI is greater than the GT, the lesion is considered to be “off-track” and at higher risk of dislocation. While the glenoid track concept has been validated on multiple imaging modalities [7, 28] and been shown to be highly predictive of recurrent instability [54], it is important to note that it remains vulnerable to small errors in measurement and does not account for patient age, activity level, tissue laxity or participation in contact sports.

The clinical relevance of the glenoid track concept was demonstrated by Shaha et. al. in a study of 57 patients treated with isolated primary BR across varying degrees of bipolar bone loss [54]. They found that 6/8 (75%) of patients with ‘neglected’ off-track HSLs had recurrent instability, compared to 4/49 (8%) of patients with on-track lesions. Similarly, Lee et. al. found that patients with *non*-engaging HSLs that were retrospectively identified as ‘neglected’ off-track lesions led to recurrent dislocation in 9/15 (60%) patients [39]. Locher et. al. found that neglected off-track lesions had 8.3 times higher odds of recurrence compared to on-track lesions when treated with isolated BR [41]. While these studies are small and retrospective in nature, they suggest that checking for arthroscopic engagement may miss some off-track lesions that would benefit from the addition of remplissage. The importance of this relatively rare group of off-track but non-engaging lesions continues to evolve, and other authors have found that patients with off-track lesions that do not engage when assessed after BR have a comparably low recurrence rate to patients with on-track lesions [46].

Although the current indications for remplissage are off-track or engaging HSLs with minimal glenoid bone loss, these indications continue to evolve. Recent series have utilized remplissage both in patients with on-track HSLs and no or minimal glenoid bone loss [16], as well as in patients with significant glenoid bone loss that would traditionally be indicated for a glenoid reconstructive procedure.

## Biomechanics

### Effect of Remplissage

The remplissage procedure is a capsulotenodesis that utilizes the posterior-superior capsule and infraspinatus tendon to fill the Hill-Sachs deficit and prevent the HSL from engaging with the glenoid rim [50]. As the capsule and infraspinatus are tied down to the defect, there is a relative medialization of their attachment point on the proximal humerus. Biomechanically, this decreases the Hill-Sachs interval while the glenoid track stays constant, thus converting the HSL from off-track to on-track and increasing the stability of the glenohumeral joint.

### Joint Stiffness

Several cadaveric studies have evaluated how adding a remplissage procedure to a traditional BR affects glenohumeral joint stiffness. Giles et. al. published the first biomechanical study that evaluated Bankart repair with remplissage (BR + R) for 30% and 45% HSLs, and found that the addition of a remplissage increased glenohumeral joint stiffness (amount of anterior joint translation after application of a constant force) by up to 74% and 207%, respectively, as compared to an unaddressed HSL [25]. Grimberg et. al. found that for a standardized 2 cm x 2 cm × 0.5 cm HSL, BR + R restored joint stiffness to 96% of normal as compared to 64% for isolated BR [27]. Elkinson et. al. demonstrated that for small (15%) and medium (30%) HSLs, remplissage was able to restore glenohumeral joint stiffness to near-native values while isolated BR did not restore normal joint stiffness or stability [18]. In a separate study, Elkinson et. al. compared multiple remplissage techniques, including medial anchor placement, anchor placement in the valley of the HSL, and anchor placement in the valley with medial suture placement; all techniques were successful in significantly increasing glenohumeral joint stiffness as compared to BR alone [17]. Other beneficial biomechanical effects have been noted following remplissage, including decreased humeral head translation in the anterior–posterior plane [22] and a more posterior-inferior humeral head apex with the arm in a position of instability [2].

### Shoulder Range of Motion

While the remplissage procedure has been shown to restore glenohumeral joint stability when performed in addition to BR, one of the main concerns is that it may result in decreased shoulder range of motion due to the non-anatomic insertion of the posterior-superior rotator cuff and tightening of the posterior shoulder capsule. Despite these concerns, results of cadaveric biomechanical studies remain mixed. Elkinson et. al. found that remplissage decreased total internal–external ROM arc by approximately 14–20 with the arm adducted to the side but had no such effect with the arm abducted to 90 [18]. Giles et. al. had similar results, with a decrease of approximately 14–17 an adducted arm and no differences as compared to the intact specimens with an abducted arm [25]. Conversely, Argintar et. al. did not observe a decrease in shoulder ROM when remplissage was added to a conventional BR [2], and Garcia et. al. found that remplissage reduced external rotation by 4, which was not significantly different from isolated BR [22].

It is possible that the alterations in shoulder ROM after remplissage may also be dependent upon factors such as surgical technique or defect size. When comparing three different remplissage techniques, Elkinson et. al. noted that anchors placed either in the valley or on the medial edge of a small (15%) HSL did not reduce shoulder external rotation, whereas anchors placed in the valley with medialized suture passage did significantly decrease external rotation [17]. However, when testing specimens with moderate (30%) HSLs, they found that all techniques significantly reduced shoulder ROM. Feng et. al. conducted a finite element analysis on several different surgical repair techniques in a computerized model of combined Bankart lesion and HSL; these techniques included isolated BR and four different combinations of BR with remplissage (medial vs. lateral anchor placement, one vs. two anchors placed) [19]. They observed that a construct with two anchors placed medially in the HSL had the least amount of humeral head displacement but also resulted in decreased ROM, though they did not quantify the resultant ROM restrictions.

While the true effect of remplissage on shoulder ROM is likely dependent upon multiple factors, surgeons performing remplissage procedures should consider HSL size and surgical technique to avoid significant alterations to shoulder ROM. In order to maximize stability while preserving ROM, current biomechanical literature supports placing suture anchors within the HSL valley and avoiding excessive tissue advancement caused by extreme medialized suture passage. However, this topic would benefit from further studies comparing multiple repair techniques in HSL of varying sizes.

### Adjunct to Free Bone Block Procedure

Though many biomechanical studies have focused on the addition of a remplissage to a soft tissue repair, a recent study by Callegari et. al. used a bipolar bone loss model to examine the effect of remplissage in the setting of a free bone block (FBB) procedure [8]. Compared to a distal tibial allograft (DTA) alone or Latarjet, they found that DTA + remplissage had the lowest amount of anterior-inferior translation, highest joint stiffness, and highest force required for dislocation. Notably, they did not evaluate the effect of combined DTA and remplissage had on shoulder range of motion.

## Outcomes

### BR + R vs Isolated BR (Table 1)

**Table 1:** Clinical Outcomes, BR + R vs. Isolated BR

Author	LOE	Recurrent Instability		Non-Instability Complications	Patient Reported Outcomes
		BR + R	BR		
Macdonald et al., 2011	I	4%(2/52)	18% (9/50)	Equivalent	Equivalent
Horinek et al., 2022	III	1/48(2.1%)	7/75(9.3%)	Equivalent	Statistically significant improvement in WOSI score for BR + R patients, equivalent for other PRO measures
Yu et al., 2023	III	0/25(0%)	1/28(3.6%)	Equivalent	Higher ROWE scores for BR + R patients, equivalent for other PRO measures
Paul et al., 2023	III	4/31(12.9%)	3/31(9.7%)	Not reported	Equivalent
Franceschi et al., 2012	III	0/25(0%)	5/25(20%)	Not reported	Favoring BR + R for 2 of 3 PRO measures
Cho et al., 2016	III	2/37(5.4%)	9/35(25.7%)	Not reported	Favoring BR + R for ROWE and VAS instability, equivalent for UCLA and VAS pain
Garcia et al., 2015	III	2/10(10%)	8/14(57.1%)	Not reported	Equivalent
Nourissat et al., 2011	II	1/15(6.7%)	1/17(5.9%)	Equivalent	Equivalent Walch-Duplay scores, but with 5 patients in BR + R group reporting posterior-superior shoulder pain with activity
Ko et al., 2016	III	0/24(0%)	5/24(20.8%)	Equivalent	Favoring BR + R for ASES, ROWE and KSSI scores

Isolated arthroscopic BR remains the most common primary shoulder stabilization surgery due to its low complications, minimally invasive technique and favorable patient reported outcomes (PROs). Despite the success of BR as a standalone procedure, recurrence rates in some series have been shown to be as high as 47 to 57%, particularly in patients with bipolar bone loss, young age or participation in collision sports [23, 33]. Comparative studies between BR + R and isolated BR have sought to determine which patients and patterns of bone loss would benefit from the addition of remplissage. In the only randomized trial comparing the two techniques, Maconald et. al. compared 108 patients, all undergoing primary stabilization surgery with engaging HSLs and subcritical GBL < 15% [42••]. The BR + R group had significantly lower rates of recurrence (4% versus 18%) and revision surgery (0% vs 12%), while PROs were equivalent between the two groups. Retrospective studies by Horinek et. al. and Yu et. al. found that rates of recurrent instability did not differ between BR + R and isolated BR, but that BR + R was associated with improved PROs and lower rates of subjective apprehension [30, 60]. While Paul et. al. found similar recurrence rates and PROs between BR + R and isolated BR, there was a nonsignificant trend towards higher revision rates in BR + R [47•]. Importantly, the BR + R group had significantly greater humeral bone loss with 84% off-track HSLs versus 4% in the isolated BR group. In the remaining six studies reviewed, five found lower recurrence rates in the BR + R groups. With regards to PROs, one study reported inferior PROs with BR + R in a portion of the outcome instruments used [20], three studies found equivalent PROs [11, 23, 45], and one study had superior PROs [37]. The current data surrounding this topic supports the addition of a remplissage to BR to decrease rates of recurrent instability, and shows that BR + R results in non-inferior or superior PROs as compared to isolated BR.

### BR + R Vs Latarjet (Table 2)

**Table 2:** Clinical Outcomes, BR + R vs. Latarjet

Author	LOE	Recurrent Instability		Non-Instability Complications		Patient Reported Outcomes
		BR + R	Latarjet	BR + R	Latarjet	
Yang et al., 2018	III	5/98(5.1%)	3/91(3.3%)	1%	12.1%	Equivalent
Maiotti et al., 2023	III	4/65(6.1%)	1/66(1.5%)	3.1%	15.2%	Trend towards superior WOSI and ROWE scores for Latarjet
Paul et al., 2023	III	4/28(14.3%)	2/43(4.7%)	Not Reported		Equivalent outcome scores, but with increased subjective instability for BR + R (50% vs 21%)
Rojas-Viada et al., 2021	III	4/69(5.8%)	1/38(2.6%)	10.5%	13.1%	Equivalent
Hurley et al., 2022	III	5/41(12.2%)	2/26(7.8%)	4.9%	7.7%	Equivalent
Horinek et al., 2022	III	1/70(1.4%)	6/188(3.2%)	0%	5.9%	Favored BR + R for all outcome measures

High failure rates after isolated arthroscopic BR in patients with subcritical glenoid bone loss have expanded the indications for BR + R (including in patients without off-track lesions), and for primary glenoid bone block procedures, most commonly arthroscopic or open Latarjet. Proponents of Latarjet cite the triple effect of the surgery and its well-documented track record in restoring stability even with large amounts of bipolar bone loss, in revision cases, and in collision athletes [35, 40]. The main disadvantage of the Latarjet procedure is the complication rate, which ranges 5–15% even at high volume institutions [6, 12].

Due to the overlap in indications between the two procedures, numerous recent studies have compared the two techniques across similar amounts of preoperative glenoid bone loss. Yang et. al. compared 189 patients treated with arthroscopic BR + R or Latarjet with similar amounts of preoperative glenoid bone loss [59]. Rates of recurrent dislocation differed but were not statistically significant (BR + R 13.3% vs Latarjet 5.5%), and patient WOSI and SANE scores were equivalent. However, in the subset of patients with > 15% glenoid bone loss, Latarjet had a significantly lower recurrence rate (6.06% vs 28.6%). Similarly, in the subanalysis of patients undergoing revision stabilization surgery, the remplissage group had significantly higher recurrence rate (34.8% vs 10.3%) and higher pain scores. Other comparative studies that utilized remplissage in the setting of ≤ 15% glenoid bone loss have demonstrated comparable recurrence rates between groups but worse PROs and higher rates of subjective instability for patients who underwent BR + R [43, 48, 51].

While the previous studies limited GBL to under 20%, and trended towards higher average GBL in the Latarjet group, Horinek et. al. performed a multicenter, international retrospective study comparing BR + R to Latarjet for primary stabilization surgery across a wide range of GBL (0–47%). Mean GBL was higher in the BR + R group as compared to the Latarjet group (12.3 vs. 7.6%), and remplissage was added regardless of the glenoid track, with only 22.9% of BR + R patients having an off-track lesion [31••]. They found no difference in recurrence of instability, with low dislocation rates in both groups and superior PROs for patients who underwent BR + R. This study was followed by another where they compared BR + R to Latarjet in 47 patients with > 15% GBL. Both groups had an average GBL of 25% GBL and had similar rates of off-track lesions. At 2 year followup, there were no recurrent dislocations in either group and equivalent PROs and satisfaction [32•].

When comparing BR + R to Latarjet in patients with subcritical (< 15%) glenoid bone loss, current studies demonstrate similar rates of recurrent, supporting the notion that BR + R may provide an equivalent outcome to Latarjet in this subset of patients [34]. However, as patients who underwent Latarjet tended to have less subjective instability and equivalent or superior PROs, surgeons should consider these trade-offs when determining an operative plan for a patient with subcritical glenoid bone loss. While current studies on BR + R vs. Latarjet in patients with critical (> 15%) glenoid bone loss are limited, BR + R appears to have equivalent or inferior outcomes to Latarjet. Thus, BR + R may not be an acceptable alternative if a significant amount of glenoid bone loss is present.

### Remplissage and Free Bone Block Procedures

While free bone block procedures are effective at reducing recurrence of glenohumeral instability, there is still a subset of patients with substantial humeral bone loss who at risk for recurrent instability despite increasing the length of the glenoid track. Calvo et. al. evaluated 51 patients with bipolar bone loss at 2 year follow-up after undergoing an arthroscopic Latarjet procedure and found that the HSL in 6 of the shoulders (11.8%) remained off-track despite surgery [9]. Additionally, while there were no postoperative dislocations, patients with a persistent off-track lesion were at higher risk of reporting recurrent subluxations (33% vs. 2.2%). While their series was relatively small, it nonetheless highlights a subset of patients with bipolar bone loss who may need additional procedures to address their HSL despite undergoing a free bone block procedure. Several articles have been published on a combined Latarjet and remplissage techinque for high-risk patients with bipolar bone loss [1, 36, 52]. Although most of these articles only contain a description of the surgical technique, the study by Abboud et. al. also included a small case series of 6 patients treated with a combined open Latarjet and arthroscopic remplissage [1]. At an average of 31 months followup, all patients had good to excellent PROs and no recurrent dislocations.

BR + R may also have a role in the treatment of recurrent instability after a failed Latarjet or Bristow procedure. Lavoue compared BR to BR + R in a cohort of patients who failed a coracoid bone block procedure and found that patients with large HSLs had better results when treated with BR + R, and that only 12% of patients overall had recurrent instability [38]. Given the lack of literature on this topic, additional studies are needed to clarify when remplissage in indicated in conjunction with or after free bone block procedures.

### Effect of Remplissage on Clinical Shoulder ROM

Despite concerns that performing a remplissage in addition to other instability procedures will limit postoperative shoulder ROM, most clinical studies tend to demonstrate no significant differences between preoperative and postoperative ROM. Garcia et. al. reported an average of 5 loss of external rotation at 5 years in a study of 50 patients who underwent BR + R for an off-track HSL [24]. A more recent case series by Scanaliato et. al. demonstrated no difference between preoperative and postoperative forward flexion, external rotation, and internal rotation after BR + R in an active-duty military population [53•]. Numerous studies have also shown that there are no significant differences in shoulder ROM when comparing BR + R to isolated BR [3, 30, 48, 60] (Table 3).

**Table 3:** Clinical ROM Outcomes, BR + R vs. Isolated BR

Author	LOE	External rotation between groups at final follow-up	Forward elevation between groups at final follow-up
Macdonald et al., 2011	I	Equivalent	Equivalent
Horinek et al., 2022	III	Equivalent	Equivalent
Yu et al., 2023	III	Equivalent	Not reported
Paul et al., 2023	III	Equivalent	Not reported
Franceschi et al., 2012	III	Equivalent	Equivalent
Cho et al., 2016	III	Equivalent	Equivalent
Nourissat et al., 2011	II	Equivalent	Equivalent
Ko et al., 2016	III	Equivalent	Not reported
Frantz et al., 2020	III	Favored Isolated BR (> 20 loss of ER in 42% of BR + R vs. 16% of isolated BR, p = 0.004)	Not reported

When comparing BR + R to Latarjet, some studies have demonstrated that BR + R can result in loss of ROM in multiple planes, including forward flexion [48], internal rotation [59], and external rotation [31••, 43•]. While the losses in forward flexion and internal rotation are typically small, Horinek et. al. reported up to 16 of external rotation difference favoring Latarjet, and Maiotti reported a 27–30% loss of external rotation after BR + R as compared to 5–9% after Latarjet. (Table 4) Nonetheless, current literature demonstrates that patients should not experience significant changes in shoulder ROM if a remplissage procedure is added to a standard BR.

**Table 4:** Clinical ROM Outcomes, BR + R vs. Latarjet

Author	LOE	External rotation between groups at final follow-up	Forward elevation between groups at final follow-up
Yang et al., 2018	III	Equivalent	Equivalent
Maiotti et al., 2023	III	Favored Latarjet (8.9% loss vs. 31.7% loss)	Not reported
Paul et al., 2023	III	Equivalent	Favored Latarjet (170 vs 164, p = 0.02)
Horinek et al., 2022	III	Favored Latarjet (4 loss for BR + R vs 19 gain for Latarjet, p < 0.001)	Equivalent

Though little evidence is available regarding risk factors for postoperative stiffness after shoulder stabilization surgery, Frantz et. al. performed a cohort analysis of 38 patients who underwent BR + R and 104 patients who underwent isolated BR to determine what factors would predict ≥ 20 external rotation deficit [21]. On multivariate analysis, they found that the addition of a remplissage was an independent predictor of ≥ 20 external rotation deficit with the elbow at 90 of abduction, but was not an independent predictor of an external rotation deficit with the elbow at the side.

### Return to Sport

Rates of return to play (RTP) typically remain high following addition of a remplissage procedure, and RTP rates for BR + R appear to be non-inferior or even superior when compared to isolated BR or Latarjet. Garcia et. al. found that 95.5% of patients undergoing BR + R returned to sport, with 81% returning to an identical level of competition. A more recent systematic review by Gouveia et. al. [26] encompassing 20 studies and 736 patients found that rates of RTP for primary BR + R ranged from 68 to 100%, though overhead athletes returned at slightly lower rates than contact athletes. While Horinek [31••] and Hughes [33] found no difference in RTP between isolated BR and BR + R, recent meta-analyses [14•, 55•] suggest that BR + R may have better RTP and similar recurrence rates when compared to both isolated BR and Latarjet.

## Indications

The best current indication for remplissage is a patient who is at increased risk for recurrent dislocation due to an engaging or off-track HSL. Based on known risk factors for recurrent glenohumeral instability, relative indications for remplissage may also include younger patients, high-risk contact athletes, and patients with high-risk on-track lesions (such as those with a low distance to dislocation). Current literature also supports the use of remplissage in patients with subcritical glenoid bone loss and an engaging/off-track HSL, as recurrence rates and PROMs appear to be largely equivalent. While critical glenoid bone loss (> 15–20%) and participation in an overhead sport are not true contraindications to BR + R, consideration of alternative procedures such as Latarjet or DTA should occur in these cases given the risk of recurrent instability, inferior PROMs, and decreased patient satisfaction with BR + R. Potential contraindications to remplissage include infraspinatus pathology and glenohumeral osteoarthritis, though these have not been studied extensively in the literature.

## Author’s Preferred Technique

Our preferred technique for arthroscopic remplissage utilizes two knotless all-suture anchors similar to the technique described by McQuivey et. al. [44]. We prefer to position the patient in the lateral decubitus position. Standard posterior, anterior, and anterior–superior portals are created with 8 mm cannulas. Prior to anterior capsulolabral repair, the HSL is viewed from the accessory superolateral portal, and the approximate size of the lesion is assessed. If necessary, an accessory posterolateral portal can be created for improved visualization of the HSL. The HSL is prepared using an elevator and a bone-cutting shaver to provide a biologically favorable surface for tissue healing. A separate posterior cannulation is made into the subacromial space. An 18G spinal needle is then placed through the cannula and through the posterior cuff approximately 5-10 mm posterior to the rent in the capsule created by the portal. A nitinol wire is placed through the spinal needle, and Seldinger technique is used to introduce a cannulated switching stick, followed by the drill guide for a 2.6 mm knotless all-suture anchors (Fibertak; Arthrex, Naples, FL). A pilot hole for the anchor is drilled in the central valley of the HSL at the border between the middle 1/3 and posterior 1/3 of the lesion, and the anchor is placed and deployed by pulling back on the sutures. An identical technique is used to introduce the drill guide 5-10 mm anterior to the capsular rent and place the second anchor between the anterior 1/3 and middle 1/3 of the lesion. At this point, all sutures have been passed through two separate areas of the infraspinatus and posterior capsule, but are all exiting through a single posterior portal within the subacromial space. The repair suture from the anterior anchor is then placed into the looped end of the conversion stitch of the posterior anchor, and the anchor is converted. The same process is repeated with the repair suture from the posterior anchor and the conversion stitch of the anterior anchor to create a suture bridge. Prior to tightening the remplissage sutures, the anterior capsulolabral repair proceeds in the standard fashion. The remplissage repair sutures are then sequentially tensioned to reduce the rotator cuff and capsule into the Hill-Sachs lesion. Following reduction, sutures are cut with a flush cutter.

## Conclusion

The addition of a remplissage procedure to a conventional arthroscopic Bankart repair in the setting of an off-track or engaging Hill-Sachs lesion results in improved shoulder biomechanics and lower rates of recurrent dislocation without significant effects on shoulder mobility. Indications for remplissage are continuing to expand and in the future may include patients with high-risk on-track Hill-Sachs lesions, ≥ 15% glenoid bone loss, or a failed prior free bone block transfer. Further high-level evidence is still required to delineate which patients may benefit the most by adding a remplissage to conventional shoulder stabilization procedures.

## Data Availability

No datasets were generated or analysed during the current study.
